# Supplementary material for: Experimental Hut Trials Reveal That CYP6P9a/b P450 Alleles Are Reducing the Efficacy of Pyrethroid-Only Olyset Net against the Malaria Vector Anopheles funestus but PBO-Based Olyset Plus Net Remains Effective
Source: Pathogens. 2022 Jun 1;11(6):638. doi: 10.3390/pathogens11060638 (PMC9228255; doi:10.3390/pathogens11060638)
Supplement: Supplementary file 1 [file pathogens-11-00638-s001.zip › pathogens-1673706-supplementary.pdf]

pathogens

MDPI

Supplementary files

Table S1. Results of the performance of Olyset and Olyset Plus against *An. funestus* females (crossing FUMOZ-FANG; F5) in experimental hut trial.

	Control	Treatments Olyset	Olyset Plus
Total	141	224	213
Exophily%	13.5	23.7*	7.5*
Blood fed (%)	8.5	11.6^{NS}	5.6^{NS}
Mortality (%)	9.9	56.7**	99.1***

ns/*: level of significance compared to control

Table S2. Correlation between *CYP6P9a and CYP6P9b* genotypes and mosquito mortality against Olyset and Olyset Plus nets after cone assays with the FANG-FUMOZ mosquito strain.

		OR	*p* value	CI	OR	P value	CI
			CYP6P9a			***CYP6P9b***	
**Olyset**	RR vs SS	35.1	< 0.00001	11.0–111.2	32.4	< 0.00001	10.8–97.3
	RS vs SS	34.6	< 0.00001	14.4–83.52	97.3	< 0.00001	34.2–277.1
	RR vs RS	0.9	1.0	0.3–2.8	3.0	> 0.05	0.9–9.4
	R vs S	7.7	< 0.001	3.9–15.2	11.1	< 0.001	5.2–23.4
**Olyset Plus**	RR vs SS	6.4	< 0.001	2.3–17.4	9.7	< 0.001	3.2–29.1
	RS vs SS	7.2	< 0.001	2.6–20.14	17.9	< 0.001	5.7–55.8
	RR vs RS	1.1	> 0.05	0.6–2.0	1.8	> 0.05	0.9–3.5
	R vs S	2.0	0.02	1.1–3.7	2.2	< 0.01	1.2–4.1

Table S3. Correlation between the *6.5Kv SV* genotypes and mosquito mortality against Olyset and Olyset Plus nets after cone assays with the FANG-FUMOZ mosquito strain.

**Olyset**		**Mortality**		
		**OR**	**PV**	**CI**
	RR vs SS	52.6	< 0.0000	17.7–161.7
	RS vs SS	27.5	< 0.00001	11.42–66.3
	RR vs RS	1.91	0.1	0.6–5.3
	R vs S	9.9	< 0.00001	5.0–19.8
	34 alive vs 17 dead			
**Olyset Plus**	RR vs SS	13	< 0.0001	4.71–35.74
	RS vs SS	8.4	< 0.0001	2.74–25.74
	RR vs RS	1.3	0.3	0.6–2.7
	R vs S	7.1	0.00001	3.4–14.8
	18 alive vs 37 dead			

Table S4. Correlation between genotypes of *CYP6P9a*, *CYP6P9b* and *6.5 kb SV* and mortality and blood-feeding after exposure to Olyset and Olyset Plus in experimental huts.

		**OR**	***p* value**	**CI**	**OR**	***p* value**	**CI**	**OR**	***p* value**	**CI**
			**Mortality**							
**Olyset**			***CYP6P9a***			***CYP6P9b***			***6.5 kb*** **ǀ*SV***	
**Unfed room**	**RR vs SS**	5.0	0.001	2.01–12.4	15.0	0.01	4.48–50.30	6.94	< 0.0001	2.8–16.7
	RS vs SS	1.8	0.1	0.8–3.9	6.0	1.0	1.9–18.75	1.6	0.2	0.7–3.6
	RR vs RS	2.7	0.7	1.6–6.08	2.4	0.006	1.2–4.8	4.2	< 0.0001	2.1–8.5
	R vs S	2.04	0.02	1.1–3.6	2.5	0.004	1.4–4.5	2.7	0.001	1.5–4.9
All samples	RR vs SS	7.03	0.009	2.5–19.4	5.1	< 0.01	1.8–14.6	6.6	0.0003	2.4–18.2
	RS vs SS	2.1	0.1	0.8–5.7	1.9	1.0	0.7–5.3	1.4	0.4	0.5–3.8
	RR vs RS	3.1	0.05	1.6–6.1	2.5	< 0.01	1.3–4.8	4.5	0.0008	2.3–8.6
	R vs S	2.4	0.004	1.3–4.3	2.4	< 0.01	1.3–4.3	2.8	0.001	1.5–5.1
			Blood feeding							
**Olyset**			***CYP6P9a***			***CYP6P9b***			***6.5 kb-SV***	
Room	RR vs SS	4.5	0.01	1.9–10.3	10.01	< 0.0001	4.1–24.9	9.6	< 0.0001	3.9–23.1
	RS vs SS	0.8	1.0	0.3–1.7	0.8	0.0008	0.3–2.2	0.9	1.0	0.4–2.2
	RR vs RS	5.4	0.001	2.6–10.9	11.1	1.0	5.3–23.03	10.2	< 0.0001	4.9–21.1
	R vs S	2.3	0.01	1.3–4.1	4.7	0.001	2.5–8.1	4.2	< 0.0001	2.3–7.9
All samples	RR vs SS	3.1	0.0005	1.2–7.8	8.01	0.01	2.4–25.9	10.01	< 0.0001	3.2–31.4
	RS vs SS	1.2	0.01	0.5–2.8	1.8	1.0	0.5–5.8	1.7	0.4	0.5–5.4
	RR vs RS	3.7	< 0.0001	1.9–7.1	4.3	0.006	2.3–8.1	5.7	< 0.0001	3.0–10.9
	R vs S	1.99	0.03	1.1–3.5	2.9	0.004	1.5–5.4	3.8	< 0.0001	2.0–7.2
Olyset Plus			*CYP6P9a*			*CYP6P9b*			*6.5 kb* ǀ*SV*	
All samples	RR vs SS	2.05	0.1	0.9–4.4				2.4	0.03	1.1–5.1
	RS vs SS	0.3	0.03	0.1–0.7				1.1	0.6	0.5–2.3
	RR vs RS	6.3	< 0.0001	3.1–12.5				2.05	0.06	1.06–3.9
	R vs S	2.0	0.03	1.1–3.5				1.6	0.1	0.9–2.8

Table S5. *CYP6P9a* and *CYP6P9b* acting together further increase the ability of *An. funestus* to survive and blood feed against Olyset after experimental hut trial.

		***CYP6P9a/b* combined**		
		Mortality all		
		OR	PV	CI
Olyset	RR/RR vs SS/SS	8.61	< 0.01	1.8–39.1
	RR/RR vs RS/SS	1.8	> 0.05	0.3–11.2
	RR/RR vs RR/SS	2.0	< 0.01	0.3–13.3
	RR/RR vs RR/RS	12.2	< 0.001	3.0–49.9
	RR/RR vs RS/RS	2.6	1.0	1.2–5.5
Olyset		Bloodfeeding all		
		OR	PV	CI
	RR/RR vs SS/SS	8.1	≤ 0.05	2.4–27.58
	RR/RR vs RS/SS	2.4	< 0.05	0.5–11.1
	RR/RR vs RR/SS	1.4	1.0	0.2–8.2
	RR/RR vs RR/RS	1.8	> 0.05	0.7–4.7
	RR/RR vs RS/RS	5.8	< 0.001	2.8–12.05
		Bloodfeeding Room only		
	RR/RR vs SS/SS	9.3	< 0.001	3.5–24.8
	RR/RR vs RS/SS	1.5	> 0.05	0.2–8.9
	RR/RR vs RR/SS	1.5	> 0.05	0.2–8.9
	RR/RR vs RR/RS	0.5	1.0	0.1–1.7
	RR/RR vs RS/RS	11.0	< 0.001	4.9–24.47
